# From Uremic Toxins to Hemodialysis Access Failure: IL-8 and MCP-1 Chemokines as a Link Between Endothelial Activation and AV Access Complications

**DOI:** 10.3390/toxins17090434

**Published:** 2025-08-31

**Authors:** Rania Chermiti, Stanislas Bataille, Philippe Giaime, Justine Solignac, Nathalie Pedinielli, Nathalie McKay, Dorian Bigey-Frau, Guillaume Lano, Hamza Benjelloun, Tawfik Addi, Julien Mancini, Stéphane Burtey, Laetitia Dou

**Affiliations:** 1 C2VN, Aix-Marseille University, INSERM, INRAE, 13385 Marseille, France; raniachermiti123@gmail.com (R.C.); stanislas.bataille@gmail.com (S.B.); justine.solignac@univ-amu.fr (J.S.); nathalie.mc-kay.1@univ-amu.fr (N.M.); dorian.bigey-frau@etu.univ-amu.fr (D.B.-F.); guillaume.lano@gmail.com (G.L.); tawfik.addi@gmail.com (T.A.); stephane.burtey@univ-amu.fr (S.B.); 2 Institut Phocéen de Néphrologie, Clinique Bouchard, ELSAN, 13005 Marseille, France; philippegiaime@icloud.com (P.G.); recherche.clinique.ipn@gmail.com (N.P.); 3 Centre de Néphrologie et Transplantation Rénale, APHM, Hôpital Conception, 13005 Marseille, France; 4 Public Health Department (BIOSTIC), APHM, Hop Timone, 13005 Marseille, France; hamza.benjelloun@ap-hm.fr (H.B.); julien.mancini@ap-hm.fr (J.M.); 5 Faculty of Natural and Life Sciences, University of Oran 1, Oran 31000, Algeria; 6 SESSTIM, Aix-Marseille University, INSERM, IRD, ISSPAM, 13005 Marseille, France

**Keywords:** arteriovenous access, chemokines, endothelial cells, hemodialysis, uremic toxins

## Abstract

Arteriovenous (AV) access complications remain a major cause of morbidity in hemodialysis patients, influenced by multiple factors, including endothelial inflammation induced by uremia. In this study, we investigated the mechanisms underlying the upregulation of endothelial chemokines interleukin-8 (IL-8) and monocyte chemoattractant protein-1 (MCP-1) by indolic uremic toxins, as well as their association with AV access complications in hemodialysis patients. In cultured human endothelial cells, IL-8 and MCP-1 were upregulated by indolic uremic toxins through activation of their receptor, the aryl hydrocarbon receptor (AHR), and non-canonical TGF-β pathway involving TAK1/p38 MAPK/AP-1 signaling. In a retrospective observational study of 204 hemodialysis patients, baseline serum IL-8 or MCP-1 were positively correlated with indolic uremic toxins and TGFβ1. Additionally, serum IL-8 ≥ 40.26 pg/mL and serum MCP-1 were independently associated with an increased risk of AV access complications over a 2-year period. In conclusion, we demonstrated that indolic uremic toxins promote endothelial inflammation by inducing IL-8 and MCP-1 expression via AHR activation and non-canonical TGF-β signaling. Clinically, elevated serum IL-8 and MCP-1 were independently associated with an increased risk of AV access complications in hemodialysis patients.

## 1. Introduction

In patients with kidney failure, effective hemodialysis depends on reliable access to the bloodstream, which can be achieved using a surgically created arteriovenous (AV) access, such as a fistula (AVF) or a synthetic graft (AVG) [1]. Complications related to vascular access remain a significant clinical issue and represent the main cause of dialysis-related morbidity, with few specific medical treatments available for prevention [1]. The main complication of AV access is stenosis, which reduces flow and can lead to thrombosis and loss of the AV access [1,2]. Stenosis in AV access is primarily caused by neointimal hyperplasia in the venous–anastomotic segment [3]. Endothelial dysfunction, inflammation, and abnormal behavior of vascular smooth muscle cells (VSMCs), characterized by their proliferation and migration into the intima, are key mechanisms underlying neointimal hyperplasia in AV access [2,3,4].

The importance of inflammatory factors in AV access complications has been highlighted by several clinical studies demonstrating an association between markers of systemic inflammation such as CRP, procalcitonin, IL-6, and an elevated monocyte-to-lymphocyte ratio, and the risk of AV access dysfunction [5,6,7]. Notably, the inverse association between AV access survival and systemic immune-inflammation indices may be useful for predicting AV access failure in clinical practice [8].

Beyond these systemic markers, more local factors, such as the endothelial chemokines interleukin-8 (IL-8/*CXCL8*) and monocyte chemoattractant protein-1 (MCP-1/*CCL2*), could be of particular interest. IL-8 and MCP-1 chemokines may not only contribute to inflammatory cell recruitment but also drive neointimal hyperplasia through their effects on VSMC proliferation and migration, leading to intimal thickening [2,9,10,11,12,13,14]. Blood levels of MCP-1 [15] and IL-8 are elevated in hemodialysis patients [16,17], and endothelial synthesis of these chemokines increases in response to the altered hemodynamic flow conditions characteristic of AV access [18]. In rodent models, MCP-1 is upregulated in the venous segment of the AVF, where it is notably localized in the endothelium and is linked to AVF outcomes [11]. MCP-1 blood levels are particularly enhanced in patients with AVF dysfunction [19] and independently predict post-angioplasty AVF restenosis [20]. Although fewer data are available on IL-8 in the context of AV access, baseline IL-8 expression in perivascular adipose tissue has been associated with early venous diameter changes in AVF [21].

Endotheliotoxic uremic toxins, which accumulate in the blood and tissues of patients with chronic kidney disease (CKD) due to impaired renal excretion, are key contributors of endothelial inflammation [22]. Notably, indolic toxins derived from dietary tryptophan, indoxyl sulfate, and indole-3 acetic acid (IAA) mediate an endothelial inflammatory and procoagulant phenotype through activation of their receptor, Aryl hydrocarbon Receptor (AHR), which in turn activates pro-inflammatory signaling pathways [23,24,25,26,27]. Indolic toxins exhibit limited clearance by hemodialysis treatment [28] due to their high degree of protein binding—approximately 90% for indoxyl sulfate and 70% for IAA [29]—while high-volume hemodiafiltration is more effective than standard low-flux dialysis in removing both the free and bound forms of indoxyl sulfate [30]. Elevated serum levels of indolic toxins are predictive of adverse cardiovascular outcomes in patients with CKD [26,31]. Indoxyl sulfate increases the expression of MCP-1 [23] and IL-8 [32,33] by endothelial cells and correlates with serum levels of MCP-1 in patients at different stages of CKD [34]. While the role of AHR in the endothelial induction of MCP-1 by indoxyl sulfate has been established [23], the mechanisms behind IL-8 upregulation by indolic toxins remain unclear.

AHR has been shown to interact with numerous kinases, including TGFβ-activated kinase 1 (TAK1) [35], a component of the non-canonical, Smad-independent, TGFβ signaling pathways [36]. The impairment of TGFβ signaling by indoxyl sulfate has been reported in non-endothelial cell types [37,38], raising the possibility of a similar effect in endothelial cells. TGFβ signaling has been associated with both AV access outcome [39,40,41] and the regulation of endothelial MCP-1 and IL-8 expression [10,42,43]. In line with this, we recently demonstrated that myostatin, a member of the TGFβ superfamily, both amplifies the upregulation of IL-8 and MCP-1 induced by indoxyl sulfate and predicts the risk of AV access complications [44]. Together, these findings suggest that endothelial expression of MCP-1 and IL-8 may be influenced by high levels of indolic toxins and TGFβ signaling, with potential implications for AV access complications.

In this study, we first investigated the signaling pathways involved in IL-8 and MCP-1 upregulation by indolic uremic toxins in cultured endothelial cells, with the aim of identifying potential therapeutic targets to mitigate endothelial inflammation. Second, we examined the association between serum concentrations of IL-8 and MCP-1 and the occurrence of flow-related complications in AV access in hemodialysis patients.

## 2. Results

### 2.1. IL-8 and MCP-1 Are Upregulated by Indolic Toxins in Cultured Endothelial Cells

**Figure 1:**
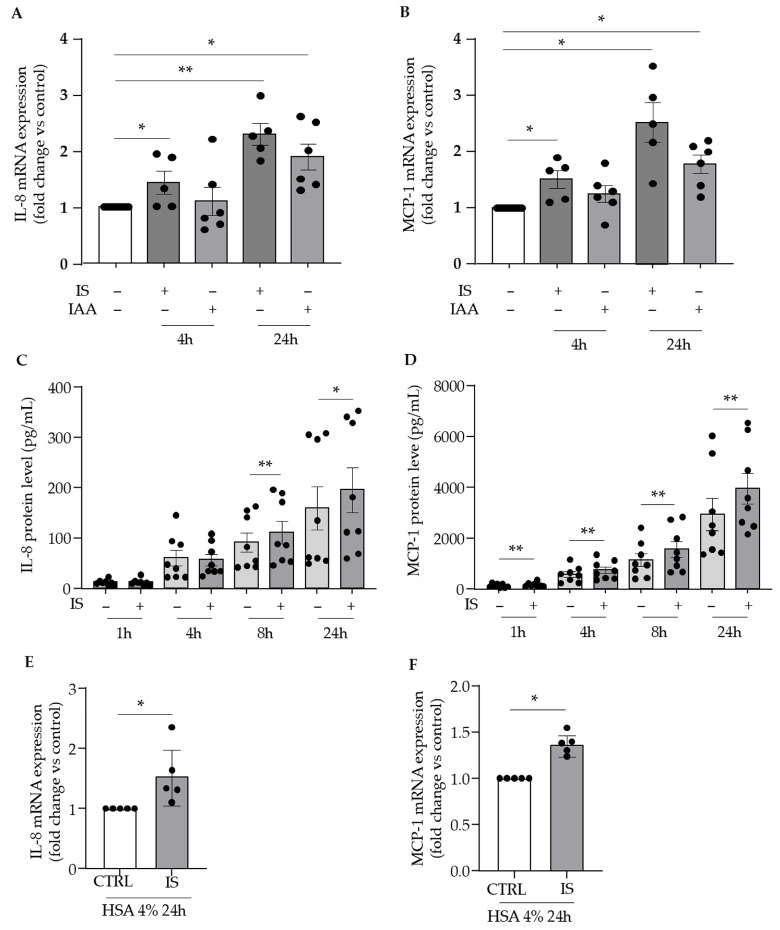
Indolic toxins upregulate IL-8 and MCP-1 in cultured endothelial cells. mRNA expression of IL-8 and MCP-1 was studied by comparative RT-qPCR and expressed in mRNA fold change vs. control (CTRL) after endothelial cell incubation with 200 µM of indoxyl sulfate (IS) or 50 µM IAA (**A**,**B**) for 4 h and 24 h. The protein levels of IL-8 (**C**) and MCP-1 (**D**) were studied by ELISA after 1 h, 4 h, 8 h, and 24 h of incubation with 200 µM indoxyl sulfate. mRNA experiments were also performed in medium supplemented with 4% of human serum albumin (**E**,**F**). Data of mRNA expression (**A**,**B**,**E**,**F**) represent the mean ± SEM of 5 independent experiments for IS and 6 independent experiments for IAA. Data of protein levels (**C**,**D**) represent the mean ± SEM of 8 independent experiments. * *p* < 0.05, ** *p* < 0.01.

We first investigated the effect of indolic uremic toxins on IL-8 and MCP-1 expression in cultured human umbilical vein endothelial cells (HUVECs). Indoxyl sulfate at 200 µM significantly upregulated both IL-8 and MCP-1 mRNA expression at 4 and 24 h (Figure 1A,B). A similar but delayed response was observed with 50 µM IAA, which induced a significant upregulation of both chemokines only at 24 h (Figure 1A,B). Indoxyl sulfate enhanced the release of IL-8 and MCP-1 proteins in HUVEC supernatants, starting from 8 and going up to 48 h for IL-8 and from as early as 1 h to up to 48 h for MCP-1 (Figure 1C,D).

Given that indolic toxins are largely protein-bound in plasma, we examined the upregulation of IL-8 and MCP-1 by indoxyl sulfate, the toxin with the highest protein-binding capacity [29], in the presence of 4 g/dL human serum albumin. Despite the presence of a physiological concentration of albumin, we still observed a significant increase in IL-8 (Figure 1E) and MCP-1 (Figure 1F) expression, indicating that the pro-inflammatory effects of indoxyl sulfate persist even under conditions where its protein binding is increased.

### 2.2. AHR Activation Is Involved in Indolic Toxin-Induced Upregulation of IL-8 and MCP-1 in Endothelial Cells

**Figure 2:**
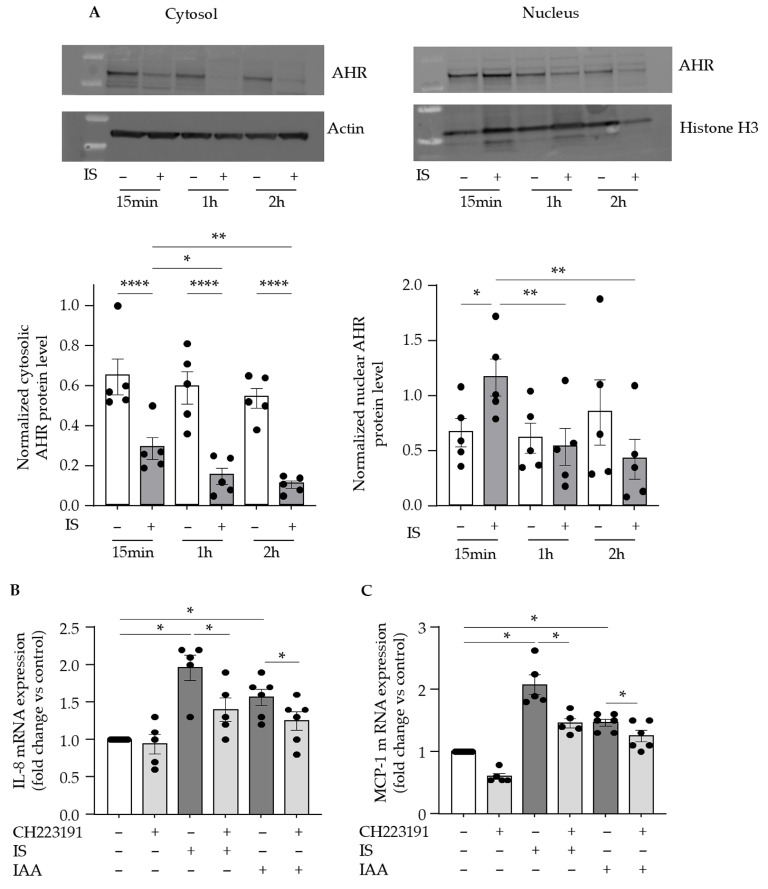
Indolic toxins indoxyl sulfate and IAA increase IL-8 and MCP-1 expression via AHR activation. (**A**) AHR levels in nuclear and cytoplasmic extracts were studied by Western blot after 15 min, 1 h, and 2 h of HUVEC stimulation with 200 µM indoxyl sulfate (IS). Pictures are representative of 5 independent experiments. mRNA expression of IL-8 (**B**) and MCP-1 (**C**) in HUVEC was studied by RT-qPCR after 24 h of stimulation with 200 µM indoxyl sulfate (IS) or 50 µM IAA in the presence of the inhibitor of AHR activation CH223191 (0.5 µM). Data expressed in mRNA fold change vs. control represent the mean ± SEM of 5 independent experiments for IS and 6 independent experiments for IAA. * *p* < 0.05, ** *p* < 0.01, **** *p* < 0.0001.

We investigated whether the receptor for indolic toxins, AHR, is involved in the upregulation of endothelial IL-8 and MCP-1. As we previously showed with IAA [27], we confirmed that indoxyl sulfate activates endothelial AHR, as evidenced by a rapid AHR nuclear translocation at 15 min (Figure 2A). After 15 min of endothelial stimulation by indoxyl sulfate, the protein level of AHR increased in the nucleus and decreased in the cytosol, indicating AHR nuclear translocation (Figure 2A). Subsequently, cytoplasmic expression of AHR protein significantly decreased after 1 h and 2 h incubation, indicating AHR degradation at these time points (Figure 2A). In the presence of CH223191, a pharmacological inhibitor of AHR activation, the upregulation of IL-8 (Figure 2B) and MCP-1 (Figure 2C) mediated by indolic toxins was significantly reduced, indicating the role of AHR activation in this upregulation.

### 2.3. Indolic Toxins Upregulate Endothelial IL-8 and MCP-1 Through Activation of the TAK1 Non-Canonical TGFβ Signaling Pathway

**Figure 3:**
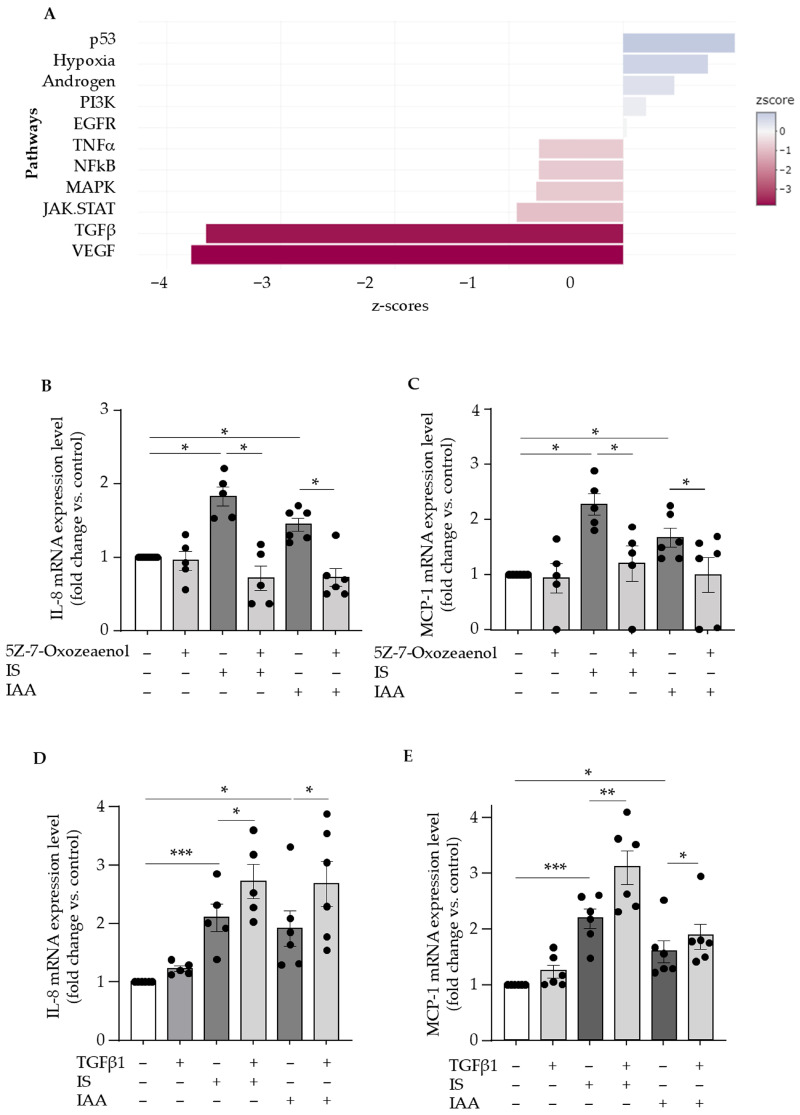
Indolic toxins upregulate IL-8 and MCP-1 by activating the TGFβ non-canonical pathway of TGF-β-activated kinase 1 (TAK1). (**A**) Dysregulated signaling pathways determined by analysis of the transcriptomic profile of endothelial cells treated for 24 h with indoxyl sulfate compared to control medium. Transcriptomic analyses were performed on RNA-Seq data previously published in the NCBI GEO database (https://www.ncbi.nlm.nih.gov/geo/query/acc.cgi?acc=GSE132410, accession number: GSE132410 accessed on 03 July 2024) by Pei et al. The z-scores of adjusted *p*-values were determined using the Progeny package. IL-8 (**B**) and MCP-1 (**C**) mRNA expression was studied by comparative RT-qPCR after 1 h of preincubation with the TAK1 inhibitor 5Z-7-oxozeaenol (OXO) at 10 µM followed by 24 h of stimulation with 200 µM indoxyl sulfate (IS) or 50 µM IAA. Data expressed in fold change vs. control represent the mean ± SEM of 5 independent experiments for IS and 6 independent experiments for IAA. * *p* < 0.05. IL-8 (**D**) and MCP-1 (**E**) mRNA expression was studied by comparative RT-qPCR in HUVEC treated for 24 h with 50 ng/mL TGFβ1 ± 200 µM indoxyl sulfate (IS) or 50 µM IAA. Data are expressed in mRNA fold change vs. control and represent the mean ± SEM of 5 (**D**) or 6 (**E**) independent experiments. * *p* < 0.05, ** *p* < 0.01, *** *p* < 0.001.

To identify signaling pathways involved in the upregulation of endothelial IL-8 and MCP-1 by indolic toxins, we analyzed RNA-Seq transcriptomic data previously published by Pei et al. in the NCBI GEO database (https://www.ncbi.nlm.nih.gov/geo/query/acc.cgi?acc=GSE132410, accession number: GSE132410 accessed on 03 July 2024), obtained from HUVECs treated for 24 h with indoxyl sulfate or control medium [45]. Our signaling pathway analysis based on z-scores of adjusted *p*-values of differentially expressed genes revealed that the TGFβ pathway was one of the most deregulated by indoxyl sulfate (Figure 3A).

Since IL-8 and MCP-1 have been identified as target genes of TGFβ signaling [10,42,43], we investigated whether indoxyl sulfate activates the canonical TGFβ pathway by testing Smad2 phosphorylation. No Smad2 phosphorylation was observed with indoxyl sulfate, in contrast to the strong response seen with TGFβ1, used as a positive control (Supplemental Figure S1). Given TGFβ also activates non-canonical, Smad-independent pathways [36], we examined the involvement of TAK1 in IL-8 and MCP-1 upregulation, as TAK1 has been shown to interact with AHR [35]. TAK1 inhibition with 5Z-7-Oxozeaenol reduced IL-8 and MCP-1 upregulation by indoxyl sulfate and IAA (Figure 3B,C), supporting the involvement of the TAK1 pathway.

We finally tested the effect of TGFβ1 on endothelial IL-8 and MCP-1 upregulation induced by indolic toxins. In the absence of toxins, TGFβ1 had no effect on IL-8 (Figure 3D) or MCP-1 (Figure 3E) expression. However, it significantly amplified the upregulation of IL-8 and MCP-1 induced by both toxins (Figure 3D,E), with the amplification of MCP-1 upregulation being less pronounced when TGFβ1 was combined with IAA than with indoxyl sulfate (Figure 3E).

### 2.4. Indolic Toxins Upregulate Endothelial IL-8 and MCP-1 via the Activation of p38 MAPK/AP-1 Signaling Pathway

**Figure 4:**
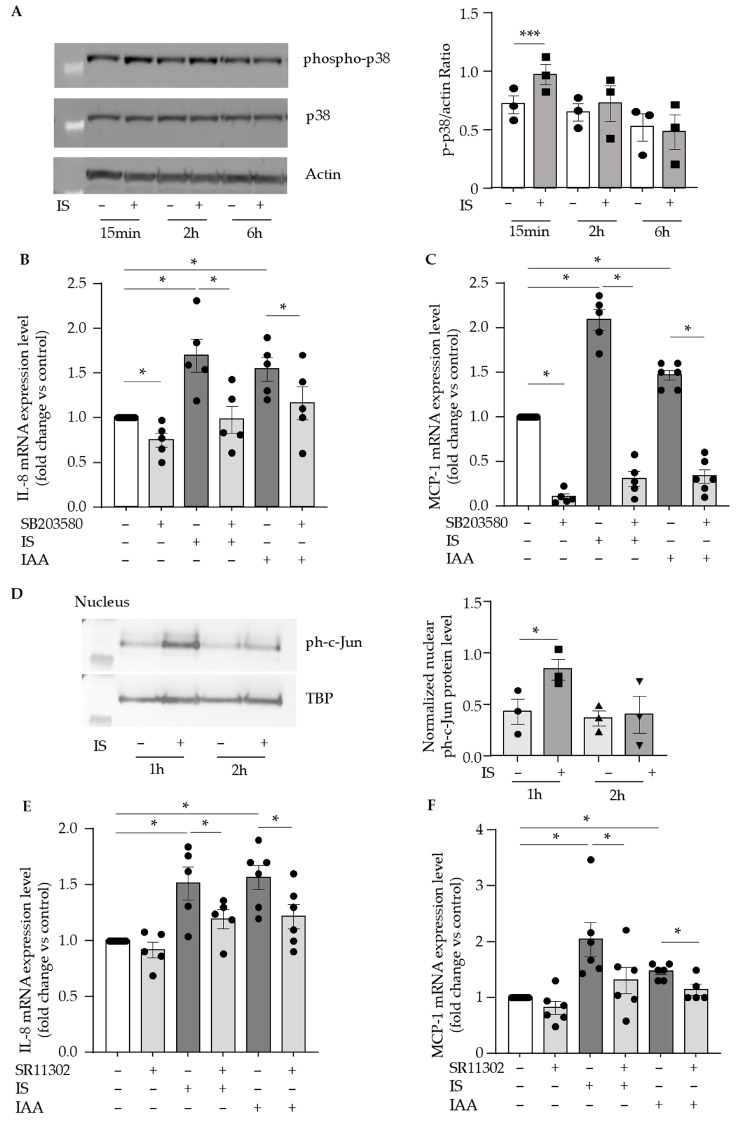
Involvement of p38MAPK/AP-1 JUN in endothelial IL-8 and MCP-1 upregulation by indolic toxins. (**A**) Phosphorylated p38 level in HUVEC protein extracts was studied by Western blot after 15 min, 2 h, and 6 h of stimulation with 200 µM indoxyl sulfate (IS). Blots are representative of 3 independent experiments. IL-8 (**B**) and MCP-1 (**C**) mRNA expression was studied by comparative RT-qPCR after 24 h of stimulation with indoxyl sulfate (200 µM) or 50 µM IAA in the presence of p38 inhibitor SB203580 (10 µM) and expressed in mRNA fold change vs. control. Data represent the mean ± SEM of 5 independent experiments. (**D**) The levels of phospho-c-Jun (ph-c-Jun) in HUVEC nuclear extracts were studied by Western blot after 15 min, 1 h, and 2 h of stimulation with 200 µM indoxyl sulfate (IS). Pictures are representative of 3 independent experiments. IL-8 (**E**) and MCP-1 (**F**) mRNA expression was studied by comparative RT-qPCR after 24 h of stimulation with 200 µM indoxyl sulfate (IS) or 50 µM IAA in the presence of the AP-1 inhibitor SR11302 (10 µM) and expressed in mRNA fold change vs. control. Data represent the mean ± SEM of 6 independent experiments. * *p* < 0.05 *** *p* < 0.001.

As TAK1 is a major upstream activator of both the p38 MAPK and canonical IκB kinase (IKK) pathways [46], we investigated their roles in IL-8 and MCP-1 upregulation by indolic toxins. As we previously showed with IAA [26], phosphorylated p38 (T180/Y182) levels were significantly increased after 15 min of indoxyl sulfate treatment (Figure 4A). IL-8 and MCP-1 upregulation by indolic toxins was significantly inhibited by the p38 inhibitor SB203580 (Figure 4B,C), suggesting a key role for the p38 MAPK pathway. In contrast, the IKK/NF-κB inhibitor BAY117082 induced a modest reduction in chemokine expression, but this effect did not reach statistical significance (Supplemental Figure S2), suggesting that NF-κB was not a major contributor.

Downstream of TAK1, activated p38 MAPK can trigger activation of the AP-1 transcription factor, which is known to regulate IL-8 and MCP-1 expression [47,48]. We analyzed the nuclear levels of the AP-1 subunit c-Jun in its phosphorylated form, ph-c-Jun, and found that they increased 1 h after indoxyl sulfate treatment (Figure 4D). Additionally, the AP-1 inhibitor SR11302 decreased IL-8 and MCP-1 upregulation (Figure 4E,F) by indoxyl sulfate and IAA, suggesting that AP-1 activation is involved in the endothelial chemokine induction by indolic uremic toxins.

### 2.5. IL-8 and MCP-1 Serum Concentrations Correlate with Indolic Toxins and TGFβ1 in Hemodialysis Patients

**Figure 5:**
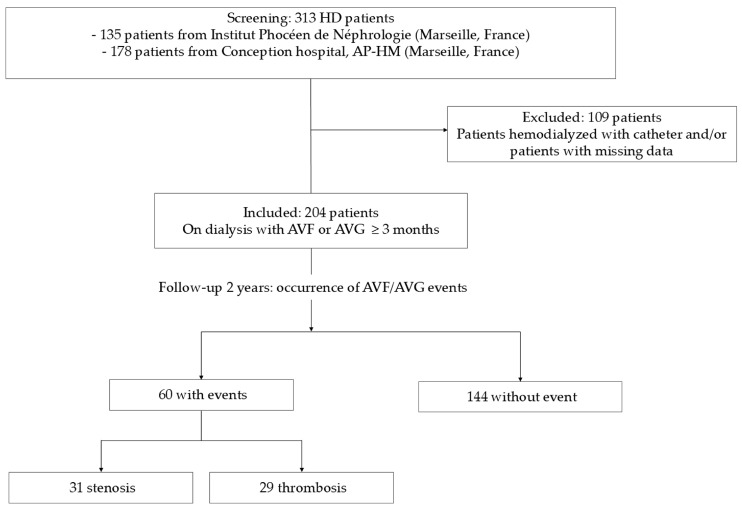
Flow chart.

We studied a cohort of 204 patients undergoing dialysis with an AV access. After a 2-year follow-up, 60 patients had an AV access event, including 31 cases of stenosis and 29 cases of thrombosis (Figure 5). During the same period, 57 patients died (15 out of 60 in the group with events, 42 out of 144 in the group without events, *p* = NS).

The baseline characteristics of the cohort are presented in Table 1.

In patients, median IL-8 serum concentration was 40.26 pg/mL (mean ± SD was 71 ± 120 pg/mL) and ranged from 0 to 1299 pg/mL (Table 1). Median MCP-1 concentration was 432 pg/mL (mean ± SD was 457 ± 175 pg/mL) and ranged from 115 to 1333 pg/mL (Table 1). Patients with IL-8 serum concentrations ≥ 40.26 pg/mL had higher systolic and diastolic blood pressure before dialysis; more history of hypertension, diabetes, coronary artery disease, and heart failure; and higher serum concentrations of IL-6, IAA, TGFβ, and lower PTH (Table 1). In Spearman correlation analysis, IL-8 serum concentrations were positively correlated with TGFβ1 (rho = 0.26; *p* = 0.0004), IAA (rho = 0.14; *p* = 0.05), IL-6 (rho = 0.22; *p* = 0.006), MCP-1 (rho = 0.18; *p* = 0.01), and sBP and dBP before dialysis (rho = 0.17, *p* = 0.02; and rho = 0.15, *p* = 0.03, respectively) (Table 2).

Spearman correlation analysis also showed that MCP-1 serum concentrations were positively correlated with indoxyl sulfate (rho = 0.22; *p* = 0.002), TGFβ1 (rho = 0.22; *p* = 0.003), parathyroid hormone (rho = 0.21; *p* = 0.003), and body mass index (rho = 0.18; *p* = 0.02), and negatively correlated with ferritin (rho = −0.21; *p* = 0.002) (Table 3). Note that MCP-1 serum concentrations were not correlated with the inflammatory markers CRP or IL-6.

### 2.6. IL-8 and MCP-1 Serum Concentrations Are Associated with Arteriovenous Access Events in Hemodialysis Patients

**Figure 6:**
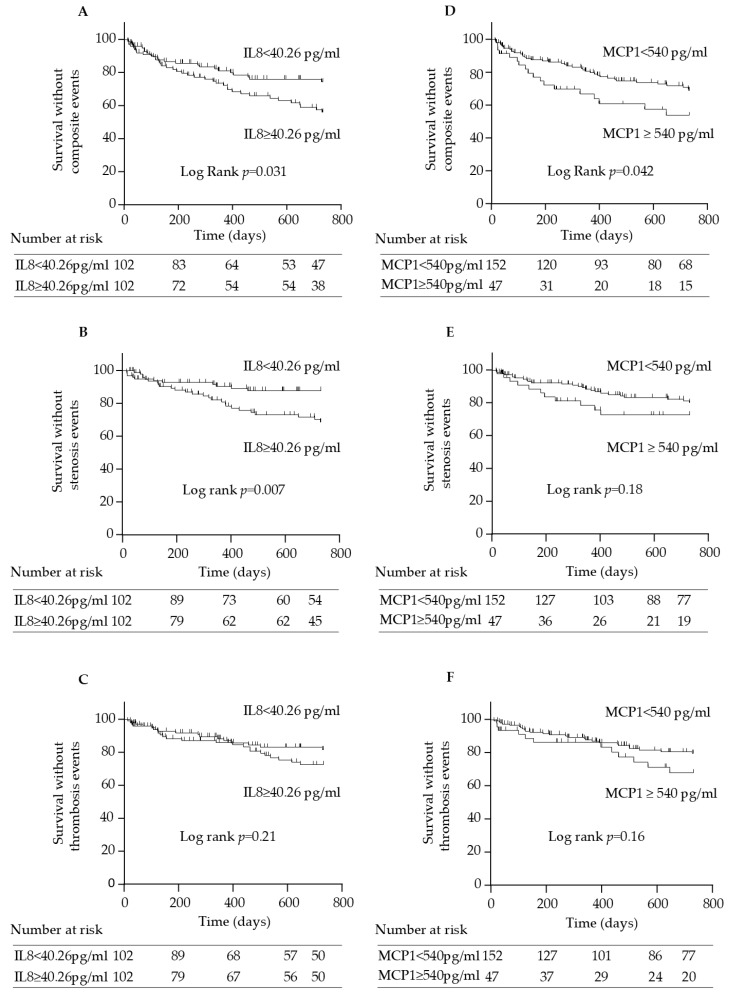
Kaplan–Meier estimates of cumulative survival without a composite event (**A**), stenosis event (**B**), or thrombosis event (**C**) of all patients according to serum IL-8 concentrations above and below the median of 40.26 pg/mL. Kaplan–Meier estimates of cumulative survival without a composite event (**D**), stenosis event (**E**), or thrombosis event (**F**) of all patients according to serum MCP-1 concentrations above and below 540 pg/mL.

As a first step, the association of IL-8 and MCP-1 with AV access complications was assessed using Kaplan–Meier survival analysis. Patients with IL-8 levels above the median value of 40.26 pg/mL experienced significantly more AV access events compared to those with IL-8 < 40.26 pg/mL (log-rank comparison of the curves: *p* = 0.031) (Figure 6A). When stenosis and thrombosis events were analyzed separately (Figure 6B,C), a significant association was observed for stenosis events (log-rank comparison of the curves: *p* = 0.007) but not for thrombosis (log-rank *p* = 0.21).

For MCP-1, Kaplan–Meier survival analysis did not reveal a significant difference when MCP-1 was dichotomized at the median. We therefore selected the lowest rounded concentration yielding a significant log-rank test, which was 540 pg/mL, as the cutoff value for Kaplan–Meier analysis. Patients with MCP-1 concentrations ≥ 540 pg/mL had higher BMI, more history of dyslipidemia, higher PTH, and lower serum ferritin (Supplemental Table S1). These patients also experienced more AV access events than those with MCP-1 concentrations < 540 pg/mL (log-rank *p* = 0.042) (Figure 6D). However, when stenosis and thrombosis events were examined separately (Figure 6E,F), the differences between patients with MCP-1 concentrations ≥ 540 pg/mL and those with MCP-1 < 540 pg/mL were no longer statistically significant (log-rank *p* = 0.21 for stenosis events; *p* = 0.16 for thrombosis events).

It is worth noting that no association was observed between serum concentrations of IL-8 or MCP-1 and overall mortality or cardiovascular events in this cohort.

We then evaluated factors associated with an increased risk of AV access events using Cox proportional hazards models. In univariate Cox analyses (Table 4), among all baseline characteristics, only serum IL-8 ≥ 40.26 pg/mL (hazard ratio [HR] = 1.76, 95% CI [1.05–2.96]; *p* = 0.033); serum MCP-1, whether dichotomized at ≥540 pg/mL (HR = 1.77, 95% CI [1.01–3.09]; *p* = 0.043) or analyzed as a continuous variable (HR = 1.23, 95% CI [1.06–1.43]; *p* = 0.006); body mass index (HR = 1.11, 95% CI [1.06–1.17]; *p* < 0.0001); and the use of antidiabetic treatments (HR = 1.76, 95% CI [1.06–2.92]; *p* = 0.029) were significantly associated with an increased risk of AV access events. Note that the distribution of IL-8 was highly skewed, and no association was found between IL-8 levels and AV access events when IL-8 was analyzed as a continuous variable. The association of history of diabetes with an increased risk of AV access events was closed to significance (HR = 1.60, 95% CI [0.97–2.66]; *p* = 0.068).

In multivariate analyses including IL-8 ≥ 40.26 pg/mL, MCP-1, demographics (gender and age > median of 71.2 years), AVG as vascular access, history of diabetes, and body mass index (BMI) as explanatory variables, IL-8 ≥ 40.26 pg/mL (HR = 1.85, 95% CI [1.04–3.29]; *p* = 0.036), MCP-1 (HR = 1.19, 95% CI [1.01–1.39]; *p* = 0.033), and BMI ≥ 30 (HR = 2.33, 95% CI [1.16–4.64]; *p* = 0.017) remained significantly associated with an increased risk of AV access events (Table 4).

## 3. Discussion

Endothelial inflammation mediated by uremia may contribute to the development of hemodialysis AV access complications [2]. In this study, we investigated the mechanisms leading to the upregulation of the chemokines IL-8 and MCP-1 in response to indolic uremic toxins as readouts of uremia-mediated endothelial inflammation. We then examined whether the elevated serum levels of MCP-1 and IL-8, frequently observed in hemodialysis patients [15,16,17], are associated with AV access complications.

Endothelial injury mediated by indoxyl sulfate has been shown to induce neointimal hyperplasia by modulating endothelial–VSMC cross-talk, thereby promoting extensive VSMC proliferation through impairment of TGFβ signaling [49]. In this context, MCP-1 appears to play a crucial role, as it mediates the migration of VSMCs toward endothelial cells in a TGFβ-dependent manner [10]. Both MCP-1 and IL-8 are implicated in the pathogenesis of intimal hyperplasia [9,12,50], which represents a central mechanism in the development of AV access stenosis. Consistent with these findings, several studies support a role of increased levels of MCP-1 and IL-8 in AVF outcome [11,20,21].

In cultured endothelial cells, we demonstrated that MCP-1 and IL-8 are upregulated by indolic uremic toxins indoxyl sulfate and IAA via a shared mechanism involving activation of the AHR and the non-canonical TGFβ signaling pathway mediated by TAK1. TAK1 is a ubiquitin-dependent kinase of IKK, MKK4/7, and MKK3/6 [46], the kinase that phosphorylates p38 MAPK [35]. In HeLa cells, non-activated AHR was shown to interact with TAK1 and MKK3/6 and to inhibit downstream p38 MAPK signaling [35]. Our results support that AHR activation enables the activation of p38 signaling via the upstream TAK1 pathway. Downstream of TAK1 and p38 MAPK, we showed that the transcription factor AP-1 c-Jun contributes to IL-8 and MCP-1 upregulation (Supplemental Figure S3). The relevance of these in vitro findings is further reinforced by studies that confirm the activation of the AHR/TAK1–p38 MAPK/AP-1 signaling pathway in vivo. AHR activation has been observed in the vessels of mice with CKD [51]; in human AVF, the TAK1 and p38 MAPK signaling pathways are significantly activated [40]; and finally, in a murine model of AVF, increased MCP-1 expression in the venous segment is accompanied by elevated AP-1 activity [11]. Disparities regarding TGFβ signaling and the effects of TGFβ1 have been reported [42], with conflicting findings particularly regarding the upregulation of endothelial MCP-1 and IL-8 [10,42,43]. In this study, we observed that TGFβ1 alone did not affect IL-8 or MCP-1 expression in endothelial cells. However, under conditions mimicking uremia, i.e., in the presence of indolic toxins, a concentration of TGFβ1 comparable to levels found in hemodialysis patients amplified the upregulation of IL-8 and MCP-1 induced by indolic toxins. Furthermore, in hemodialysis patients, we found that MCP-1 and IL-8 levels positively correlated with TGFβ1, as well as with the indolic toxins indoxyl sulfate and IAA, respectively. These findings suggest that a context of high levels of indolic uremic toxins, as seen in hemodialysis patients, may shift TGFβ signaling towards a pro-inflammatory endothelial phenotype that mediates overexpression of MCP-1 and IL-8. In AVF, increased TGF-β signaling has been demonstrated in both humans and mice with CKD [52], and is associated with endothelial-to-mesenchymal transition, enhanced smooth muscle cell proliferation, increased AVF wall thickness, and reduced AVF patency [39,52]. Although we did not find a direct link between the levels of individual indolic uremic toxins and AV access complications in patients, our in vitro study suggests that these toxins, by disrupting TGFβ signaling, may nevertheless participate in impaired AV access outcomes, along with many other factors.

In hemodialysis patients, AHR activation has been well-documented as a result of the accumulation of tryptophan-derived uremic toxins [51,53,54]. Aside from endothelial inflammation, AHR activation has also been shown to promote thrombosis through tissue factor overexpression in endothelial cells and VSMCs [24,27,54,55,56]. This mechanistic link is supported by clinical data indicating that patients with AVF thrombosis exhibit higher AHR activity than those without thrombosis [54]. Therefore, targeting AHR activation could represent an effective strategy to reduce both endothelial inflammation and thrombosis, particularly in AV access. Additionally, beyond reducing endothelial inflammation driven by uremic toxins, inhibition of endothelial TGFβ signaling may improve AVF patency by attenuating VSMC proliferation, reducing wall thickness and enhancing outward remodeling of the AVF, as demonstrated in a mouse model [39].

The mechanisms we identified for MCP-1 and IL-8 upregulation through AHR-mediated activation of TGFβ TAK-1 pathway may also apply to other AHR agonists present in hemodialysis patients, such as kynurenines [53]. This hypothesis is consistent with the lack of correlation observed between IL-8 and indoxyl sulfate (or MCP-1 and IAA) in patients, which suggests that chemokine upregulation may be influenced by agonists other than the indolic toxins. Additionally, the overactivation of the TGFβ pathway induced by both TGFβ1 and AHR that leads to IL-8 and MCP-1 amplification might extend to other members of the TGFβ family, not just TGFβ1. Indeed, we previously observed that myostatin, a member of the TGFβ family, exerts a similar effect to TGFβ1 by amplifying indoxyl sulfate-mediated chemokine upregulation [44]. Interestingly, we also showed that myostatin is associated with an increased risk of arteriovenous access complications [44]. Taken together, these findings suggest a broader role for AHR activation and TGFβ family members in promoting inflammatory chemokine expression, which may help explain why individual indolic toxins or TGFβ1 were not associated with AV access complications in our study, whereas IL-8 and MCP-1 were.

In line with previous reports showing that serum MCP-1 levels are elevated in patients with AVF dysfunction and indicating that an increase in MCP-1 levels is associated with AVF restenosis [19,20], we observed a significant association between MCP-1 levels, whether analyzed as a continuous variable or dichotomized at ≥540 pg/mL, and the risk of AV access complications, defined as a composite of stenosis and thrombotic events. This association remained significant after adjustment for demographics, vascular access type, diabetes history, and body mass index. Although the magnitude of the risk associated with MCP-1 was modest, it reached strong statistical significance and persists when adjusted with confounding factors. Additionally, Kaplan–Meier survival analysis did not reveal a significant difference when MCP-1 was dichotomized at the median, but only when the cutoff of MCP-1 was set at 540 pg/mL, a value that also predicts the risk of AV access complications. This suggests that MCP-1 levels above this cutoff provide greater prognostic utility in identifying patients at increased risk of AV access events. Overall, the independent relationship between MCP-1 levels and the risk of AV access complications further supports the hypothesis of a pathogenic role for MCP-1 in these outcomes.

To date, studies investigating IL-8 in the context of AV access are scarce. Only one prior study has reported a link between IL-8 expression in perivascular adipose tissue and adaptative vascular remodeling in AVF [21]. In Kaplan–Meier survival analysis, we found that patients with IL-8 levels above the median value of 40.26 pg/mL experienced more AV access events. IL-8 levels above the median were also associated with an increased risk of AV access complications and remained independently predictive of the risk after adjustment for MCP-1 levels, demographics, vascular access type, diabetes history, and body mass index. The lack of association when IL-8 was modeled as a continuous variable may reflect the highly skewed distribution of IL-8 levels and suggests that only patients with particularly elevated IL-8 may be at increased risk. Our study is, to our knowledge, the first to demonstrate an independent association between circulating IL-8 levels and flow-related AV access complications, underscoring its potential as a relevant predictive biomarker in this setting.

When stenosis and thrombosis events were analyzed separately, IL-8 and MCP-1 levels were not associated with thrombosis in the AV access, despite previous reports linking both chemokines to venous thrombosis in the general population [57]. Additionally, although studies involving large patient cohorts or patients at high risk of thrombosis [54,58] have reported associations between serum indoxyl sulfate and AVF thrombosis [54], as well as its predictive value for post-angioplasty thrombosis of dialysis grafts [58], we found no association between indolic toxin levels and AV access complications. These discrepancies may be explained by the low number of thrombosis cases in our study, which represents a limitation.

In the analysis of separate outcomes, IL-8 was specifically associated with stenosis events in the AV access, while MCP-1 was linked only to the composite outcome. This suggests that IL-8 may play a greater role in stenosis development than in thrombotic processes within AV access. Both MCP-1 and IL-8 are involved in the pathogenesis of intimal hyperplasia [9,12,50], which is a key mechanism in AV access stenosis, as well as in atherosclerotic plaque formation [59], where intimal hyperplasia also plays a major role. However, although patients with elevated IL-8 and MCP-1 levels had more atherosclerosis risk factors (hypertension, coronary artery disease, diabetes for IL-8; dyslipidemia and elevated BMI for MCP-1), no association was observed between these cytokines and cardiovascular events during follow-up. This apparent discrepancy may reflect underlying differences in the pathophysiological mechanisms of neointimal hyperplasia in AV access compared to those involved in atherosclerosis. While both processes involve intimal thickening, atherosclerotic lesions are typically accompanied by lipid accumulation, atheroma, and calcification [3,4], features not commonly found in AV access stenosis. Additionally, exposure of endothelial cells to the specific hemodynamic flow conditions encountered in AV access may promote the release of greater amounts of MCP-1 and IL-8, which can stimulate VSMC proliferation, as demonstrated in a prior study [18]. Our findings suggest that, in the context of CKD, IL-8- and MCP-1-driven mechanisms may be more closely related to localized vascular remodeling/neointimal hyperplasia specific to AV access rather than to the multifactorial processes of atherosclerosis.

Given the several limitations of our study, the results should be interpreted with caution. A limitation of our in vitro model is that we assessed the effect of indoxyl sulfate in the presence of a physiological serum albumin concentration only at the level of IL-8 and MCP-1 mRNA expression. Although the effect persisted under this condition, its magnitude was reduced. Further studies are therefore needed to evaluate how IL-8 and MCP-1 protein levels, as well as the associated signaling pathways, are affected under conditions that more closely reflect the protein binding of indolic toxins in vivo. Regarding our clinical study, it was conducted on a relatively small sample of 204 patients, resulting in a limited number of events (57 deaths and 60 AV access events, including 31 stenosis and 29 thrombosis events). We defined AV access complications using a composite endpoint. However, the limited number of events and the use of a composite endpoint may have masked risk factors that are significantly associated with specific AV access events. Additionally, this is a retrospective analysis with non-randomized patient selection, restricted to two centers within the same city. Finally, we observed an association between IL-8 levels and AV access events when IL-8 was dichotomized at the median, but not when analyzing IL-8 as a continuous variable. This suggests a non-linear association, potentially indicating that only patients with IL-8 levels exceeding a certain threshold are at increased risk. However, the lack of association in the continuous analyses may have limited the study’s power and may represent a limitation.

## 4. Conclusions

In conclusion, we demonstrate that AHR activation by indolic uremic toxins disrupts TAK1-related TGFβ signaling, inducing endothelial inflammation through overexpression of IL-8 and MCP-1 chemokines. Additionally, our study identifies IL-8 and MCP-1 as potential predictors of flow-related AV access complications. Measuring IL-8 and MCP-1 levels could help identify hemodialysis patients at higher risk, enabling earlier interventions. These findings pave the way for targeting AHR/TGFβ signaling activation to mitigate endothelial inflammation and potentially improve outcomes in hemodialysis patients.

## 5. Materials and Methods

### 5.1. Patients

We conducted a multi-center retrospective observational study in two cohorts of hemodialysis patients enrolled in June 2014 in Marseille, France, and followed up with them for 2 years. One cohort (n = 135) [60] was recruited from the Institut Phocéen de Néphrologie (Clinique Bouchard, ELSAN), and the other cohort (n = 178) [61] was recruited from Conception Hospital (AP-HM). Inclusion criteria were patients older than 18 years who had been undergoing hemodialysis for more than 3 months and who gave their non-opposition to the study. Exclusion criteria included non-French speakers, those unable to give informed consent, and patients under 18. From these cohorts, we selected 204 hemodialysis patients with either an AVF (84% of patients) or an AVG (16%) as their current VA access, in whom IL-8 assays on tube bottoms were feasible (Figure 1).

Clinical and biological features, comorbidities, and treatments were collected. History of hypertension was defined as history of high blood pressure requiring treatment for more than 6 months. History of diabetes was defined as history of hyperglycemia requiring treatment for more than 6 months. History of coronary artery disease was defined as history of coronary artery disease on coronary angiography requiring at least medical treatment. History of heart failure was defined as the presence of left ventricular dysfunction on transthoracic echocardiography. History of atrial fibrillation was defined as a history of atrial fibrillation documented on electrocardiogram. History of peripheral arterial disease was defined as history of limb arterial atherosclerotic lesions on arteriography requiring revascularization. History of stroke or transient ischemic attack were defined as a history of focal neurologic deficit with or without ischemic brain lesions on imaging, respectively. History of deep vein thrombosis or pulmonary embolism was defined as a history of thrombosis documented at deep vein thrombosis or pulmonary embolism documented by imaging. History of dyslipidemia was defined as impaired lipid profile requiring treatment for more than 6 months.

The primary outcome was the incidence of AV access events, defined as a composite of the first occurrence of AV access thrombosis or clinically significant stenosis (more than 50% vessel lumen reduction) requiring endovascular treatment. Endovascular treatment was decided in both centers when flow reduction exceeded 20%, hemodialysis quality evaluated by KT/V decreased, or compression time increased.

During the study period, clinical events, including overall mortality and cardiovascular events (cardiovascular death, non-fatal myocardial infarction, non-fatal stroke, and non-fatal peripheral arterial disease with amputation or need for angioplasty) were also recorded. According to French law, it was not necessary or possible to obtain approval from an ethics committee (Comité de Protection des Personnes) for this type of non-interventional study. Moreover, Comités de Protection des Personnes ethics committees are not entitled to issue waivers of approval for this type of study. Informed consent was obtained from all individual participants included in the study.

### 5.2. Laboratory Tests in Patients

Standard laboratory procedures were used for blood chemistry evaluations at inclusion. Serum levels of uremic toxins indoxyl sulfate, indole-3 acetic acid (IAA), and p-cresyl sulfate were measured by high-performance liquid chromatography as described [62]. IL-8, MCP-1, TGFβ1, and IL-6 serum levels were measured using ELISA kits (Human IL-8/CXCL8 Quantikine ELISA, Human CCL2/MCP-1 Quantikine ELISA, Human/Mouse/Rat/Porcine/Canine TGF-β1 Quantikine ELISA, and Human IL-6 Quantikine ELISA, respectively) from R&D systems (Bio-Techne, Noyal Châtillon sur Seiche, France).

### 5.3. Endothelial Cell Culture and Treatment

Human umbilical vein endothelial cells (HUVECs) were obtained from Lonza (Colmar, France) and were grown up to the 5th passage in Endothelial Cell Growth Medium-2 (EGM2, Lonza, France) under standard culture conditions (humidified atmosphere, 37 °C, 5% CO_2_). Experiments were performed on HUVEC replicates from different cell preparations. HUVECs were treated with indolic toxin indoxyl sulfate (Merck-Sigma-Aldrich Chimie, Saint-Quentin Fallavier, France) or IAA (Merck-Sigma-Aldrich Chimie, Saint-Quentin Fallavier, France) at concentrations found in hemodialysis patients, i.e., 200 µM and 50 µM, respectively [63]. Indoxyl sulfate was diluted 1/1000 from a stock solution of indoxyl sulfate potassium salt at 200 mM. KCl diluted 1/1000 was used as a control of indoxyl sulfate. IAA was diluted from a stock solution of 50 mM prepared in ethanol. Ethanol diluted 1/1000 was used as a control of IAA. In some experiments, human serum albumin (Octapharma, Boulogne-Billancourt, France), at the concentration found in human serum (4 g/dL), was added to the medium. Cells were also treated for 24 h with TGFβ1 (Abcam, Cambridge, UK) at 50 ng/mL (a concentration found in our cohort) diluted 1/1000 from a stock solution at 50 µg/mL, or water diluted 1/1000 as control, in the presence or not of indolic toxins: indoxyl sulfate at 200 µM or IAA at 50µM. HUVECs were also treated with 200 µM indoxyl sulfate or 50 µM IAA, or control vehicles in Endothelial Cell Growth Basal Medium-2 (EBM2, Lonza, France) containing 2% fetal bovine serum (Dominique Dutscher, Bernolsheim, France), in the presence of the AHR inhibitor CH223191 (Merck-Sigma-Aldrich Chimie, Saint-Quentin Fallavier, France) at 0.5 µM, the p38 inhibitor SB203580 (Cell Signaling Technology, Ozyme, Saint-Cyr-l’Ecole, France) at 10 µM, the AP-1 inhibitor SR11302 (Bio-Techne, Noyal Châtillon sur Seiche, France) at 10 µM, and the NFκB inhibitor BAY11702 (Merck-Sigma-Aldrich Chimie, France) at 10 µM. To inhibit TAK1 activity, HUVECs were preincubated with the irreversible TAK1 inhibitor 5Z-7-oxozeaenol (Merck-Sigma-Aldrich Chimie, Saint-Quentin Fallavier, France) at 10 µM for 1 h and then treated for 24 h with 200 µM indoxyl sulfate or 50 µM IAA, or control vehicles in EBM2 medium containing 2% fetal bovine serum. These experiments were carried out in a medium that was as neutral as possible (EBM2 medium containing 2% fetal bovine serum), without additives or HSA, to specifically observe the inhibition of signaling pathways activated solely by the indolic toxins.

### 5.4. mRNA Extraction and Quantitative RT-PCR Analysis

Total RNA was extracted using the RNeasy mini kit (Qiagen, Courtaboeuf, France) after cell lysis with RLT buffer supplemented with 1% β-mercaptoethanol. Reverse transcription (RT) was performed on 500 ng of total RNA using the Takara PrimeScript™ RT reagent Kit (Takara, Saint-Germain-en-Laye, France) followed by quantitative polymerase chain reaction (qPCR) using the Takara SYBR qPCR Premix Ex Taq (Takara, Saint-Germain-en-Laye, France). We quantified the following target genes: *CCL2* (MCP-1), *CXCL8* (IL-8), and the housekeeping gene *HPRT1*, which was used to normalize the target gene values. The sequences of primers are displayed in Supplemental Table S2. All PCR reactions were performed with the Applied Biosystems Step One Plus Real-Time PCR system (Thermo Fisher Scientific, Illkirch, France). The transcript for the housekeeping gene *HPRT1* was used for data normalization. The fold change of mRNA expression versus control condition was calculated using the 2^−ΔΔCt^ method.

### 5.5. Protein Extraction and Western Blot Analysis

HUVECs were lysed with lysis buffer containing Triton X100, SDS, and protease and phosphatase inhibitors (Thermo Fisher Scientific, Illkirch, France) and centrifuged at 12,000 rpm for 15 min at 4 °C. The supernatants containing protein extracts were collected and stored at −80 °C. Protein concentration was measured with the Bicinchoninic Acid Kit for Protein Determination (BCA1, Merck, Sigma-Aldrich Chimie, Saint-Quentin Fallavier, France).

Nuclear and cytosolic extracts were prepared using the Nuclear Extraction Kit from Abcam (France), according to the manufacturer’s instructions. Cytoplasmic and nuclear proteins were measured using the Bradford reagent (Abcam, Cambridge, UK) and stored at −80 °C until use.

Equal amounts of proteins from total cell lysates or nuclear or cytoplasmic extracts were mixed with a denaturing buffer containing 4X NuPAGE-LDS (Thermo Fisher Scientific, Illkirch, France), β-mercaptoethanol, and lysis buffer. Samples were incubated at 95 °C for 5 min, loaded on 4–12% SDS-polyacrylamide electrophoresis gel, and transferred into a nitrocellulose membrane. Nonspecific binding was blocked with 5% non-fat milk at room temperature for one hour. The membrane was incubated with primary antibodies directed against AHR (Santa Cruz Biotechnology, CliniSciences, Nanterre, France), phospho-p38 (Thr180/Tyr182), p38, phospho-c-Jun (Ser73), phospho-Smad2 (Ser465/Ser467), Smad2, β-actin, histone H3, or TATA box-binding protein (TBP) (all from Cell Signaling Technology, Ozyme, Saint-Cyr-l’Ecole, France), and then with secondary peroxidase-conjugated goat anti-mouse or anti-rabbit antibodies (Thermo Fisher Scientific, Illkirch, France). Revelation was made by chemiluminescence using ECL Western blotting substrate (Thermo Fisher Scientific, Illkirch, France). The gel image was captured using the Image Quant LAS4000 (GE Healthcare, Buc, France). Densitometry analyses were performed with the Fiji (Fiji Is Just ImageJ) software, version 1.54f.

### 5.6. Study of IL-8 and MCP-1 Release in Supernatants

Cells were incubated with 200 µM indoxyl sulfate in EBM2 medium supplemented with 2% fetal bovine serum for 1 h, 4 h, 8 h, 24 h, and 48 h. Control cells were treated with KCL 1/1000. IL-8 and MCP-1 levels were measured in supernatants using the human IL-8/CXCL8 and the CCL2/MCP-1 Quantikine ELISA kits (R&D systems, Bio-Techne, Noyal Châtillon sur Seiche, France), according to the manufacturer’s instructions. Absorbance values at 450 nm were measured using a GloMax^®^ Explorer Multimode Microplate Reader (Promega, Charbonnières-les-Bains, France).

### 5.7. Study of Signaling Pathways from Transcriptomic Analyses

Analyses of signaling pathways were obtained from transcriptomic analyses performed on previously published RNA-Seq data [45]. Transcriptomic analyses were performed on sequencing data previously published in the NCBI GEO database (https://www.ncbi.nlm.nih.gov/geo/query/acc.cgi?acc=GSE132410 accessed on 3 July 2024). The accession number is GSE132410, associated with the paper by Pei J, Juni R, and Harakalova et al. published in 2019 in Toxins [45]. Differential expression analysis of the transcripts was conducted using RStudio (version 4.3.3), leveraging the limma (version 3.58.1) and DESeq2 (version 1.42.1) packages. To adjust the *p*-value, we employed the Benjamini–Hochberg procedure to calculate the false discovery rate. Subsequently, we utilized the FUNKI application through the Shiny package (version 1.8.1.1) for pathway analyses. This application facilitates the use of the Progeny package. Calculation of the z-score was carried out using the adjusted *p*-value obtained earlier, as previously described. All mentioned packages are accessible on Bioconductor (https://www.bioconductor.org/); version 3.19 was used for analyses in this study.

### 5.8. Statistical Analyses

In patients, continuous variables are expressed as median [min; max]. The Spearman rank correlation coefficient was estimated to study the relationship between variables. The Kaplan–Meier method was used to study the AV access event-free survival. The log rank test was used to compare survival distributions.

Univariate and multivariate analyses of AV access events were performed using a Cox proportional hazard model with serum IL-8 ≥ 40.26 pg/mL or serum MCP-1, analyzed either as a continuous variable or dichotomized at ≥540 pg/mL, as explanatory variables. In multivariate analyses, demographics (gender and age > median of 71.2 years), AVG as vascular access, and factors associated with AV access events in our cohort of hemodialysis patients (body mass index and history of diabetes) were added to the model. All tests were two-sided and considered statistically significant at *p* < 0.05. All analyses were performed using R 4.2 (R Foundation for Statistical Computing, Vienna, Austria).

In in vitro experiments and statistical analyses were performed with the Prism software, version 10.5.0 (GraphPad Inc., CA, USA). Significant differences were revealed by the Wilcoxon signed-rank test, by the Mann–Whitney *t*-test, or by ANOVA, followed by an uncorrected Fischer’s test, depending on experiments. Data are expressed as mean ± SEM of independent experiments performed on different cell preparations. A *p*-value < 0.05 was considered significant.

## Figures and Tables

**Table 1:** Baseline characteristics of the hemodialysis population (n = 204).

	All Patients (n = 204)	IL8< 40.26 pg/mL (n = 102)	IL8≥ 40.26 pg/mL (n = 102)	*p*-Value
Age (years)	71.2 [18; 94]	71.1 [20; 94]	71.5 [18; 94]	0.391
Gender ratio (F/M)	75/129	37/65	38/64	>0.999
Body mass index (kg/m^2^)	24.7 [12.9; 41.5]	24.5[12.9; 35.5]	24.8[16.4; 41.5]	0.211
Arteriovenous graft	33 (16%)	19 (19%)	14 (14%)	0.447
SBP before dialysis (mmHg)	140 [83; 210]	135 [84; 208]	144 [83; 210]	**0.005**
DBP before dialysis (mmHg)	70 [40; 119]	69 [40; 115]	71 [42; 119]	**0.060**
Dialysis vintage (months)	43 [3; 432]	37 [3; 421]	48 [3; 432]	0.657
History of hypertension	172 (84%)	80 (78%)	92 (90%)	**0.033**
History of diabetes	80 (39%)	30 (29%)	50 (49%)	**0.006**
History of CAD	68 (33%)	26 (25%)	42 (41%)	**0.025**
History of heart failure	45 (22%)	16 (16%)	29 (28%)	**0.042**
History of atrial fibrillation	60 (29%)	29 (28%)	31 (30%)	0.878
History of PAD	50 (24%)	20 (20%)	30 (29%)	0.145
History of stroke/TIA	28 (14%)	14 (14%)	14 (14%)	>0.999
History of DVT/PE	25 (12%)	14 (14%)	11 (11%)	0.670
History of renal transplantation	19 (9%)	8 (8%)	11 (11%)	0.630
History of dyslipidemia	60 (29%)	25 (25%)	35 (34%)	0.166
Antihypertensive drugs	137 (67%)	68 (67%)	69 (68%)	>0.999
Antidiabetic treatments	76 (37%)	29 (28%)	47 (46%)	**0.013**
Antiplatelet drugs	103 (50%)	48 (47%)	55 (54%)	0.409
Anticoagulant drugs	38 (18%)	17 (17%)	21 (21%)	0.590
Hypolipidemic drugs	60 (29%)	26 (25%)	34 (33%)	0.282
Erythropoiesis stimulating agents	154 (75%)	83 (81%)	71 (70%)	0.072
Hemoglobin (g/dL)	10.8 [6.1; 13.7]	10.6 [6.1; 13.3]	10.9 [8.0; 13.7]	0.327
Serum albumin (g/L)	38.8 [21.6; 52.1]	38.0 [23.3; 47.2]	39.0 [21.6; 52.1]	0.265
Parathyroid hormone (ng/L)	27 [1; 2217]	37 [1; 2217]	19 [2; 656]	**0.045**
Serum ferritin (ng/mL)	385 [26; 5000]	395 [26; 5000]	347 [26; 1625]	0.486
Serum calcium (mmol/L)	2.34 [1.84; 3.09]	2.33 [1.84; 3.09]	2.34 [1.84; 2.85]	0.360
Serum phosphate (mmol/L)	1.50 [0.38; 4.03]	1.45 [0.65; 4.03]	1.59 [0.38; 3.73]	0.310
Serum potassium (mmol/L)	5.01 [2.91; 7.20]	5.05 [2.91; 6.82]	5.00 [3.39; 7.2]	0.644
Serum indoxyl sulfate (µM)	87.8 [0; 301]	88.6 [0; 276]	86.3 [0; 301]	0.698
Serum indole-3 acetic acid (µM)	3.1 [0.2; 33.5]	2.7 [0.2; 22.2]	3.8 [1.3; 33.5]	**0.002**
Serum p-cresyl sulfate (µM)	148 [0; 1227]	144 [0; 1227]	153 [8; 438]	0.394
Serum CRP (mg/L)	6.3 [0.2; 418.9]	7.5 [0.2; 107.3]	5.4 [0.6; 418.9]	0.461
Serum IL-6 (pg/mL)	4 [0; 232]	2.75 [0; 92.1]	5.3 [0; 232.2]	**0.014**
Serum MCP-1 (pg/mL)	432 [112; 1333]	425 [153; 1333]	444 [115; 930]	0.069
Serum TGFβ1 (ng/mL)	22.5 [6.9; 51.8]	20.1 [8.0; 51.8]	24.7 [6.9; 39.8]	**0.004**
Serum IL-8 (pg/mL)	40.26 [0; 1299]	26.3 [0; 40.25]	73.8 [40.26; 1299]	**<0.0001**

For categorical variables, results are given as absolute counts (%). For continuous ones, results are given as median [min; max]. SBP: systolic blood pressure, DBP: diastolic blood pressure, CAD: coronary artery disease, DVT/PE: deep vein thrombosis/pulmonary embolism, PAD: peripheral arterial disease, TIA: transient ischemic attack.

**Table 2:** Spearman correlations of baseline characteristics with IL-8 serum concentrations.

Variable	Rho	*p*-Value
Serum TGFβ1	0.26	0.0004
Serum IL-6	0.22	0.006
Serum MCP-1	0.18	0.01
Serum IAA	0.14	0.05
Systolic blood pressure before dialysis	0.17	0.02
Diastolic blood pressure before dialysis	0.15	0.03

**Table 3:** Spearman correlations of baseline characteristics with MCP-1 serum concentrations.

Variable	Rho	*p*-Value
Serum indoxyl sulfate	0.22	0.002
Serum TGFβ1	0.22	0.003
Serum parathyroid hormone	0.21	0.003
Body mass index	0.18	0.02
Serum ferritin	−0.21	0.002

**Table 4:** Univariate and multivariate Cox analysis of risk factors for AV access events.

	Hazard Ratio	HR 95% CI	*p*-Value
**Univariate analysis**			
IL-8 ≥ 40.26 pg/mL	1.76	[1.05–2.96]	0.033
MCP-1 ≥ 540 pg/mL	1.77	[1.01–3.09]	0.043
MCP-1 (per 100 pg/mL increase)	1.23	[1.06–1.43]	0.006
Body mass index (BMI)	1.11	[1.06–1.17]	<0.0001
History of diabetes	1.60	[0.97–2.66]	0.068
Antidiabetic treatments	1.76	[1.06–2.92]	0.029
**Multivariate analysis**			
IL-8 ≥ 40.26 pg/mL	1.85	[1.04–3.29]	**0.036**
MCP-1 (per 100 pg/mL increase)	1.19	[1.01–1.39]	**0.033**
Gender	1.26	[0.71–2.21]	0.492
Age > 71.2 years	0.82	[0.47–1.44]	0.393
AVG	1.43	[0.68–3.02]	0.346
Normal BMI 18.5–25 (Ref)			0.082
BMI < 18.5	0.68	[0.15–3.01]	0.612
BMI 25–29.9	1.33	[0.68–2.61]	0.407
BMI ≥ 30	2.33	[1.16–4.64]	**0.017**
History of diabetes	1.20	[0.67–2.16]	0.541

## Data Availability

The data presented in this study are available on request from the corresponding author due to privacy restrictions. Transcriptomic analyses were performed on sequencing data previously published in the NCBI GEO database (https://www.ncbi.nlm.nih.gov/geo/query/acc.cgi?acc=GSE132410) under accession number GSE132410 by Pei et al. (Pei J, Juni R, Harakalova M, et al. Indoxyl Sulfate Stimulates Angiogenesis by Regulating Reactive Oxygen Species Production via CYP1B1. *Toxins (Basel).* 2019;11(8):454. doi: 10.3390/toxins11080454).

## Supplementary Materials

The following supporting information can be downloaded at: https://www.mdpi.com/article/10.3390/toxins17090434/s1, Table S1: Patient characteristics according to MCP1 serum concentrations above and below 540 pg/mL (n = 199); Table S2: Sequences of primers used in RT-qPCR experiments; Figure S1: Study of TGFβ canonical pathway activation in endothelial cells treated by indoxyl sulfate or TGFβ1; Figure S2: Study of NF-κB involvement in indoxyl sulfate-induced MCP-1 and IL8 expression in HUVECs; Figure S3. Signaling pathways potentially involved in IL-8 and MCP-1 upregulation by indolic toxins.

## References

[1] Lok C.E., Huber T.S., Orchanian-Cheff A., Rajan D.K. (2024). Arteriovenous Access for Hemodialysis: A Review. JAMA.

[2] Ma S., Duan S., Liu Y., Wang H. (2022). Intimal Hyperplasia of Arteriovenous Fistula. Ann. Vasc. Surg..

[3] Rothuizen T.C., Wong C., Quax P.H.A., van Zonneveld A.J., Rabelink T.J., Rotmans J.I. (2013). Arteriovenous Access Failure: More than Just Intimal Hyperplasia?. Nephrol. Dial. Transplant..

[4] Roy-Chaudhury P., Sukhatme V.P., Cheung A.K. (2006). Hemodialysis Vascular Access Dysfunction: A Cellular and Molecular Viewpoint. J. Am. Soc. Nephrol..

[5] Zhang Y., Yi J., Zhang R., Peng Y., Dong J., Sha L. (2022). Risk Factors for Arteriovenous Fistula Thrombus Development: A Systematic Review and Meta-Analysis. Kidney Blood Press. Res..

[6] Zhang F., Yu J., Li G., Fu S., Xiao H., Yang Y., Liang Y., Chen Y., Luo X. (2024). The Risk Factors for Arteriovenous Fistula Dysfunction in Maintenance Hemodialysis Patients: A Cross-Sectional Study. Hemodial. Int..

[7] Ciucanu C.C., Mureșan A., Florea E., Réka B., Mureșan A.V., Szanto L.-A., Arbănași E.-M., Hosu I., Russu E., Arbănași E.-M. (2025). Elevated Interleukin-6 Is Associated with an Increased Risk of Long-Term Arteriovenous Fistula Failure for Dialysis. J. Clin. Med..

[8] Ren S., Xv C., Wang D., Xiao Y., Yu P., Tang D., Yang J., Meng X., Zhang T., Zhang Y. (2024). The Predictive Value of Systemic Immune-Inflammation Index for Vascular Access Survival in Chronic Hemodialysis Patients. Front. Immunol..

[9] Zeller I., Knoflach M., Seubert A., Kreutmayer S.B., Stelzmüller M.E., Wallnoefer E., Blunder S., Frotschnig S., Messner B., Willeit J. (2010). Lead Contributes to Arterial Intimal Hyperplasia through Nuclear Factor Erythroid 2-Related Factor-Mediated Endothelial Interleukin 8 Synthesis and Subsequent Invasion of Smooth Muscle Cells. Arterioscler. Thromb. Vasc. Biol..

[10] Ma J., Wang Q., Fei T., Han J.-D.J., Chen Y.-G. (2007). MCP-1 Mediates TGF-Beta-Induced Angiogenesis by Stimulating Vascular Smooth Muscle Cell Migration. Blood.

[11] Juncos J.P., Grande J.P., Kang L., Ackerman A.W., Croatt A.J., Katusic Z.S., Nath K.A. (2011). MCP-1 Contributes to Arteriovenous Fistula Failure. J. Am. Soc. Nephrol..

[12] Qin Y., Fan F., Zhao Y., Cui Y., Wei X., Kohama K., Gordon J.R., Li F., Gao Y. (2013). Recombinant Human CXCL8(3-72)K11R/G31P Regulates Smooth Muscle Cell Proliferation and Migration through Blockage of Interleukin-8 Receptor. IUBMB Life.

[13] Spinetti G., Wang M., Monticone R., Zhang J., Zhao D., Lakatta E.G. (2004). Rat Aortic MCP-1 and Its Receptor CCR2 Increase with Age and Alter Vascular Smooth Muscle Cell Function. Arterioscler. Thromb. Vasc. Biol..

[14] Yue T.L., Wang X., Sung C.P., Olson B., McKenna P.J., Gu J.L., Feuerstein G.Z. (1994). Interleukin-8. A Mitogen and Chemoattractant for Vascular Smooth Muscle Cells. Circ. Res..

[15] Papayianni A., Alexopoulos E., Giamalis P., Gionanlis L., Belechri A.-M., Koukoudis P., Memmos D. (2002). Circulating Levels of ICAM-1, VCAM-1, and MCP-1 Are Increased in Haemodialysis Patients: Association with Inflammation, Dyslipidaemia, and Vascular Events. Nephrol. Dial. Transplant..

[16] Lisowska K.A., Storoniak H., Soroczyńska-Cybula M., Maziewski M., Dębska-Ślizień A. (2022). Serum Levels of α-Klotho, Inflammation-Related Cytokines, and Mortality in Hemodialysis Patients. J. Clin. Med..

[17] Nakanishi I., Moutabarrik A., Okada N., Kitamura E., Hayashi A., Syouji T., Namiki M., Ishibashi M., Zaid D., Tsubakihara Y. (1994). Interleukin-8 in Chronic Renal Failure and Dialysis Patients. Nephrol. Dial. Transplant..

[18] Franzoni M., Cattaneo I., Longaretti L., Figliuzzi M., Ene-Iordache B., Remuzzi A. (2016). Endothelial Cell Activation by Hemodynamic Shear Stress Derived from Arteriovenous Fistula for Hemodialysis Access. Am. J. Physiol. Heart Circ. Physiol..

[19] De Marchi S., Falleti E., Giacomello R., Stel G., Cecchin E., Sepiacci G., Bortolotti N., Zanello F., Gonano F., Bartoli E. (1996). Risk Factors for Vascular Disease and Arteriovenous Fistula Dysfunction in Hemodialysis Patients. J. Am. Soc. Nephrol..

[20] Wu C.-C., Chen T.-Y., Hsieh M.-Y., Lin L., Yang C.-W., Chuang S.-Y., Tarng D.-C. (2017). Monocyte Chemoattractant Protein-1 Levels and Postangioplasty Restenosis of Arteriovenous Fistulas. Clin. J. Am. Soc. Nephrol..

[21] Mauro C.R., Ding K., Xue H., Tao M., Longchamp A., Belkin M., Kristal B.S., Ozaki C.K. (2016). Adipose Phenotype Predicts Early Human Autogenous Arteriovenous Hemodialysis Remodeling. J. Vasc. Surg..

[22] Chermiti R., Burtey S., Dou L. (2024). Role of Uremic Toxins in Vascular Inflammation Associated with Chronic Kidney Disease. J. Clin. Med..

[23] Watanabe I., Tatebe J., Namba S., Koizumi M., Yamazaki J., Morita T. (2012). Activation of Aryl Hydrocarbon Receptor Mediates Indoxyl Sulfate-Induced Monocyte Chemoattractant Protein-1 Expression in Human Umbilical Vein Endothelial Cells. Circ. J..

[24] Gondouin B., Cerini C., Dou L., Sallée M., Duval-Sabatier A., Pletinck A., Calaf R., Lacroix R., Jourde-Chiche N., Poitevin S. (2013). Indolic Uremic Solutes Increase Tissue Factor Production in Endothelial Cells by the Aryl Hydrocarbon Receptor Pathway. Kidney Int..

[25] Lano G., Laforêt M., Von Kotze C., Perrin J., Addi T., Brunet P., Poitevin S., Burtey S., Dou L. (2020). Aryl Hydrocarbon Receptor Activation and Tissue Factor Induction by Fluid Shear Stress and Indoxyl Sulfate in Endothelial Cells. Int. J. Mol. Sci..

[26] Dou L., Sallee M., Cerini C., Poitevin S., Gondouin B., Jourde-Chiche N., Fallague K., Brunet P., Calaf R., Dussol B. (2015). The Cardiovascular Effect of the Uremic Solute Indole-3 Acetic Acid. J. Am. Soc. Nephrol..

[27] Addi T., Poitevin S., McKay N., El Mecherfi K.E., Kheroua O., Jourde-Chiche N., de Macedo A., Gondouin B., Cerini C., Brunet P. (2019). Mechanisms of Tissue Factor Induction by the Uremic Toxin Indole-3 Acetic Acid through Aryl Hydrocarbon Receptor/Nuclear Factor-Kappa B Signaling Pathway in Human Endothelial Cells. Arch. Toxicol..

[28] Duval-Sabatier A., Burtey S., Pelletier M., Laforet M., Dou L., Sallee M., Lorec A.-M., Knidiri H., Darbon F., Berland Y. (2023). Systematic Comparison of Uremic Toxin Removal Using Different Hemodialysis Modes: A Single-Center Crossover Prospective Observational Study. Biomedicines.

[29] Deltombe O., Van Biesen W., Glorieux G., Massy Z., Dhondt A., Eloot S. (2015). Exploring Protein Binding of Uremic Toxins in Patients with Different Stages of Chronic Kidney Disease and during Hemodialysis. Toxins.

[30] Panichi V., Rocchetti M.T., Scatena A., Rosati A., Migliori M., Pizzarelli F., Gesualdo L., REDERT Study group (2017). Long Term Variation of Serum Levels of Uremic Toxins in Patients Treated by Post-Dilution High Volume on-Line Hemodiafiltration in Comparison to Standard Low-Flux Bicarbonate Dialysis: Results from the REDERT Study. J. Nephrol..

[31] Barreto F.C., Barreto D.V., Liabeuf S., Meert N., Glorieux G., Temmar M., Choukroun G., Vanholder R., Massy Z.A. (2009). Serum Indoxyl Sulfate Is Associated with Vascular Disease and Mortality in Chronic Kidney Disease Patients. Clin. J. Am. Soc. Nephrol..

[32] Bouabdallah J., Zibara K., Issa H., Lenglet G., Kchour G., Caus T., Six I., Choukroun G., Kamel S., Bennis Y. (2019). Endothelial Cells Exposed to Phosphate and Indoxyl Sulphate Promote Vascular Calcification through Interleukin-8 Secretion. Nephrol. Dial. Transplant..

[33] Savira F., Kompa A.R., Magaye R., Xiong X., Huang L., Jucker B.M., Willette R.N., Kelly D.J., Wang B.H. (2021). Apoptosis Signal-Regulating Kinase 1 Inhibition Reverses Deleterious Indoxyl Sulfate-Mediated Endothelial Effects. Life Sci..

[34] Borges N.A., Barros A.F., Nakao L.S., Dolenga C.J., Fouque D., Mafra D. (2016). Protein-Bound Uremic Toxins from Gut Microbiota and Inflammatory Markers in Chronic Kidney Disease. J. Ren. Nutr..

[35] Li H., Hong W., Jin X., Li G., Zhou G., Fan L. (2019). The Aryl Hydrocarbon Receptor Is a Novel Negative Regulator of Interleukin-17-Mediated Signaling and Inflammation in Vitro. FEBS Lett..

[36] Tzavlaki K., Moustakas A. (2020). TGF-β Signaling. Biomolecules.

[37] Shimizu H., Yisireyili M., Nishijima F., Niwa T. (2013). Indoxyl Sulfate Enhances P53-TGF-Β1-Smad3 Pathway in Proximal Tubular Cells. Am. J. Nephrol..

[38] Lekawanvijit S., Kompa A.R., Manabe M., Wang B.H., Langham R.G., Nishijima F., Kelly D.J., Krum H. (2012). Chronic Kidney Disease-Induced Cardiac Fibrosis Is Ameliorated by Reducing Circulating Levels of a Non-Dialysable Uremic Toxin, Indoxyl Sulfate. PLoS ONE.

[39] Taniguchi R., Ohashi Y., Lee J.S., Hu H., Gonzalez L., Zhang W., Langford J., Matsubara Y., Yatsula B., Tellides G. (2022). Endothelial Cell TGF-β (Transforming Growth Factor-Beta) Signaling Regulates Venous Adaptive Remodeling to Improve Arteriovenous Fistula Patency. Arterioscler. Thromb. Vasc. Biol..

[40] Hu H., Lee S.-R., Bai H., Guo J., Hashimoto T., Isaji T., Guo X., Wang T., Wolf K., Liu S. (2020). TGFβ (Transforming Growth Factor-Beta)-Activated Kinase 1 Regulates Arteriovenous Fistula Maturation. Arterioscler. Thromb. Vasc. Biol..

[41] Stracke S., Konner K., Köstlin I., Friedl R., Jehle P.M., Hombach V., Keller F., Waltenberger J. (2002). Increased Expression of TGF-Beta1 and IGF-I in Inflammatory Stenotic Lesions of Hemodialysis Fistulas. Kidney Int..

[42] Lux A., Salway F., Dressman H.K., Kröner-Lux G., Hafner M., Day P.J.R., Marchuk D.A., Garland J. (2006). ALK1 Signalling Analysis Identifies Angiogenesis Related Genes and Reveals Disparity between TGF-Beta and Constitutively Active Receptor Induced Gene Expression. BMC Cardiovasc. Disord..

[43] Wu X., Ma J., Han J.-D., Wang N., Chen Y.-G. (2006). Distinct Regulation of Gene Expression in Human Endothelial Cells by TGF-Beta and Its Receptors. Microvasc. Res..

[44] Solignac J., Dou L., Chermiti R., McKay N., Giaime P., Pedinielli N., Benjelloun H., Lano G., Mancini J., Burtey S. (2025). Myostatin Exacerbates Endothelial Dysfunction Induced by Uremic Toxin Indoxyl Sulfate and Is Associated with Hemodialysis Arteriovenous Access Complications. Toxins.

[45] Pei J., Juni R., Harakalova M., Duncker D.J., Asselbergs F.W., Koolwijk P., van Hinsbergh V., Verhaar M.C., Mokry M., Cheng C. (2019). Indoxyl Sulfate Stimulates Angiogenesis by Regulating Reactive Oxygen Species Production via CYP1B1. Toxins.

[46] Wang C., Deng L., Hong M., Akkaraju G.R., Inoue J., Chen Z.J. (2001). TAK1 Is a Ubiquitin-Dependent Kinase of MKK and IKK. Nature.

[47] Srinivasan S., Bolick D.T., Hatley M.E., Natarajan R., Reilly K.B., Yeh M., Chrestensen C., Sturgill T.W., Hedrick C.C. (2004). Glucose Regulates Interleukin-8 Production in Aortic Endothelial Cells through Activation of the P38 Mitogen-Activated Protein Kinase Pathway in Diabetes. J. Biol. Chem..

[48] Ito S., Osaka M., Edamatsu T., Itoh Y., Yoshida M. (2016). Crucial Role of the Aryl Hydrocarbon Receptor (AhR) in Indoxyl Sulfate-Induced Vascular Inflammation. J. Atheroscler. Thromb..

[49] Ryu J.H., Park H., Kim S.J. (2017). The Effects of Indoxyl Sulfate-Induced Endothelial Microparticles on Neointimal Hyperplasia Formation in an Ex Vivo Model. Ann. Surg. Treat. Res..

[50] Fu C., Yu P., Tao M., Gupta T., Moldawer L.L., Berceli S.A., Jiang Z. (2012). Monocyte Chemoattractant Protein-1/CCR2 Axis Promotes Vein Graft Neointimal Hyperplasia through Its Signaling in Graft-Extrinsic Cell Populations. Arterioscler. Thromb. Vasc. Biol..

[51] Dou L., Poitevin S., Sallée M., Addi T., Gondouin B., McKay N., Denison M.S., Jourde-Chiche N., Duval-Sabatier A., Cerini C. (2018). Aryl Hydrocarbon Receptor Is Activated in Patients and Mice with Chronic Kidney Disease. Kidney Int..

[52] Zhang W., Gonzalez L., Li X., Bai H., Li Z., Taniguchi R., Langford J., Ohashi Y., Thaxton C., Aoyagi Y. (2024). Endothelial TGF-β Signaling Regulates Endothelial-Mesenchymal Transition During Arteriovenous Fistula Remodeling in Mice with Chronic Kidney Disease. Arterioscler. Thromb. Vasc. Biol..

[53] Sallée M., Dou L., Cerini C., Poitevin S., Brunet P., Burtey S. (2014). The Aryl Hydrocarbon Receptor-Activating Effect of Uremic Toxins from Tryptophan Metabolism: A New Concept to Understand Cardiovascular Complications of Chronic Kidney Disease. Toxins.

[54] Kolachalama V.B., Shashar M., Alousi F., Shivanna S., Rijal K., Belghasem M.E., Walker J., Matsuura S., Chang G.H., Gibson C.M. (2018). Uremic Solute-Aryl Hydrocarbon Receptor-Tissue Factor Axis Associates with Thrombosis after Vascular Injury in Humans. J. Am. Soc. Nephrol..

[55] Chitalia V.C., Shivanna S., Martorell J., Balcells M., Bosch I., Kolandaivelu K., Edelman E.R. (2013). Uremic Serum and Solutes Increase Post-Vascular Interventional Thrombotic Risk through Altered Stability of Smooth Muscle Cell Tissue Factor. Circulation.

[56] Addi T., Dou L., Burtey S. (2018). Tryptophan-Derived Uremic Toxins and Thrombosis in Chronic Kidney Disease. Toxins.

[57] van Aken B.E., den Heijer M., Bos G.M., van Deventer S.J., Reitsma P.H. (2000). Recurrent Venous Thrombosis and Markers of Inflammation. Thromb. Haemost..

[58] Wu C.C., Hsieh M.Y., Hung S.C., Kuo K.L., Tsai T.H., Lai C.L., Chen J.W., Lin S.J., Huang P.H., Tarng D.C. (2016). Serum Indoxyl Sulfate Associates with Postangioplasty Thrombosis of Dialysis Grafts. J. Am. Soc. Nephrol..

[59] Munjal A., Khandia R. (2020). Atherosclerosis: Orchestrating Cells and Biomolecules Involved in Its Activation and Inhibition. Adv. Protein Chem. Struct. Biol..

[60] Bataille S., Landrier J.-F., Astier J., Giaime P., Sampol J., Sichez H., Ollier J., Gugliotta J., Serveaux M., Cohen J. (2016). The “Dose-Effect” Relationship Between 25-Hydroxyvitamin D and Muscle Strength in Hemodialysis Patients Favors a Normal Threshold of 30 Ng/mL for Plasma 25-Hydroxyvitamin D. J. Ren. Nutr..

[61] Lano G., Sallée M., Pelletier M., Bataille S., Fraisse M., Berda-Haddad Y., Brunet P., Burtey S. (2019). Mean Platelet Volume Predicts Vascular Access Events in Hemodialysis Patients. J. Clin. Med..

[62] Calaf R., Cerini C., Genovesio C., Verhaeghe P., Jourde-Chiche N., Berge-Lefranc D., Gondouin B., Dou L., Morange S., Argiles A. (2011). Determination of Uremic Solutes in Biological Fluids of Chronic Kidney Disease Patients by HPLC Assay. J. Chromatogr. B Anal. Technol. Biomed. Life Sci..

[63] Vanholder R., De Smet R., Glorieux G., Argiles A., Baurmeister U., Brunet P., Clark W., Cohen G., De Deyn P.P., Deppisch R. (2003). Review on Uremic Toxins: Classification, Concentration, and Interindividual Variability. Kidney Int..

