# Supplementary material for: From Uremic Toxins to Hemodialysis Access Failure: IL-8 and MCP-1 Chemokines as a Link Between Endothelial Activation and AV Access Complications
Source: Toxins (Basel). 2025 Aug 31;17(9):434. doi: 10.3390/toxins17090434 (PMC12473946; doi:10.3390/toxins17090434)

Supplemental Tables

Supplemental Table S1. Patient's characteristics according to MCP1 serum concentrations above and below 540 pg/ml (n=199)

	MCP1< 540 pg/ml (n=152)	MCP1> 540 pg/ml (n=47)	p
Age (years)	70.8 [18; 94]	70.8 [23; 94]	0.607
Gender ratio (F/M)	55/97	19/28	0.608
Body Mass Index (kg/m²)	24.4 [13.0; 39.5]	26.0 [17.1; 41.5]	**0.049**
Arteriovenous graft	25 (16%)	7 (15%)	>0.999
SBP before dialysis (mmHg)	140 [87; 210]	140 [83; 177]	0.415
DBP before dialysis (mmHg)	70 [41; 119]	69 [40; 99]	0.430
Dialysis vintage (months)	51 [3; 421]	35 [3; 432]	0.717
History of hypertension	126 (83%)	41 (87%)	0.650
History of diabetes	56 (37%)	21 (45%)	0.392
History of CAD	51 (34%)	15 (32%)	>0.999
History of heart failure	34 (22%)	9 (19%)	0.691
History of atrial fibrillation	43 (28%)	16 (34%)	0.468
History of PAD	37 (24%)	13 (28%)	0.701
History of stroke/TIA	20 (13%)	8 (17%)	0.481
History of DVT/PE	20 (13%)	4 (9%)	0.456
History of renal transplantation	15 (10%)	4 (9%)	>0.999
History of dyslipidemia	38 (25%)	21 (45%)	**0.017**
Antihypertensive drugs	105 (69%)	29 (62%)	0.376
Antidiabetic treatments	53 (35%)	21 (45%)	0.232
Antiplatelet drugs	76 (50%)	26 (55%)	0.617
Anticoagulant drugs	28 (18%)	9 (19%)	>0.999
Hypolipidemic drugs	44 (29%)	14 (30%)	>0.999
Erythropoiesis stimulating agents	110 (72%)	39 (83%)	0.179
Hemoglobin (g/dl)	10.8 [6.1; 13.7]	10.7 [8.3; 13.4]	0.617
Serum CRP (mg/l)	6.1 [0.2; 113.7]	8.2 [0.6; 418.9]	0.125
Serum IL-6 (pg/ml)	5 [0; 232]	4 [0; 124]	0.413
Parathyroid hormone (ng/l)	21 [1; 2217]	49 [2; 657]	**0.013**
Serum ferritin (ng/ml)	428 [26; 5000]	278 [49; 1551]	**0.032**
Serum calcium (mmol/l)	2.34 [1.84; 3.09]	2.34 [1.99; 2.76]	0.960
Serum phosphate (mmol/l)	1.48 [0.38; 3.73]	1.53 [0.73; 4.03]	0.924
Serum potassium (mmol/l)	5.03 [2.91; 7.2]	5.00 [3.76; 6.82]	0.297
Serum indoxyl sulfate (μM)	86.2 [0; 240.8]	100.8 [26.3; 301]	0.158
Serum indole-3 acetic acid (μM)	3.1 [0.2; 33.5]	3.3 [1.1; 19.7]	0.902
Serum p-cresyl sulfate (μM)	149 [0; 559]	133 [15; 1227]	0.535
Serum CRP (mg/l)	6.1 [0.2; 113.7]	8.2 [0.6; 418.9]	0.125
Serum IL-6 (pg/ml)	5 [0; 232]	4 [0; 124]	0.413
Serum TGFβ1 (ng/ml)	22.0 [6.9; 48.3]	24.6 [10.0; 51.8]	0.073
Serum IL-8 (pg/ml)	38 [0; 1299]	47 [12; 370]	0.108

For categorical variables, results are given as absolute counts (%). For continuous ones, results are given as median [min; max]. SBP: systolic blood pressure, DBP: diastolic blood pressure, CAD: coronary artery disease, DVT/PE: deep vein thrombosis/pulmonary embolism, PAD: peripheral arterial disease, TIA: Transient ischemic attack.

Supplemental Table S2. Sequences of primers used in RT-qPCR experiments

Gene	Primer forward	Primer reverse
CCL2	5′ TCTGTGCCTGCTGCTCATAG 3′	5′ CAGATCTCCTTGGCCACAAT 3′
CXCL8	5′ CTCCACAACCCTCTGCAC 3 ′	5′ TGCCAAGGAGTGCTAAAG 3′
HPRT	5′ GGATTATACTGCCTGACCAAGGAAAGC 3′	5′ GAGCTATTGTAATGACCAGTCAACAGG 3′

Supplemental Figures

Supplemental Figure S1. Study of TGFβ canonical pathway activation in endothelial cells treated by indoxyl sulfate or TGFβ1. Phosphorylated Smad2 level in protein extracts was studied by Western blot after 15min and 6h of HUVEC stimulation with indoxyl sulfate (IS 200 µM) or TGFβ1 (50 ng/mL), used as positive control. Pictures are representative of 3 independent experiments.

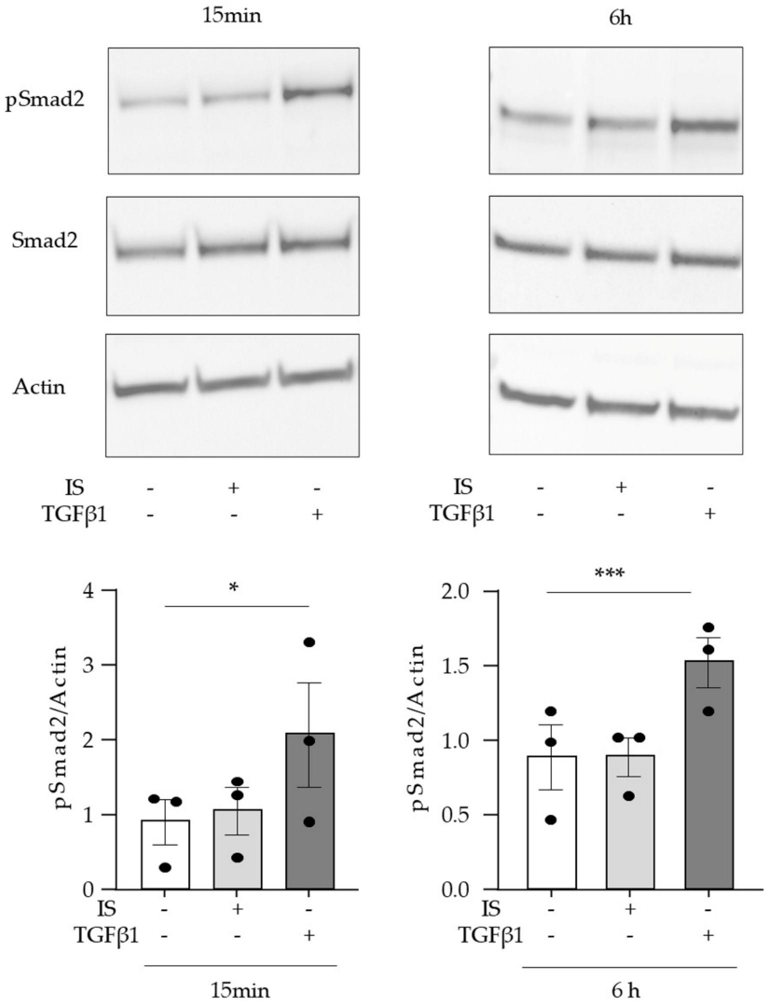

Supplemental Figure S2. Study of NF-κB involvement in indoxyl sulfate-induced MCP-1 and IL8 expression in HUVEC. MCP-1 (A) and IL-8 (B) mRNA expression was studied by comparative RT-qPCR after 4h of stimulation with indoxyl sulfate (IS 200 μM) in the presence of the NF-κB inhibitor BAY117082 (10 μM). Results are expressed in mRNA fold change vs. control. Data represent the mean ± SEM of 5 independent experiments.

A **B**

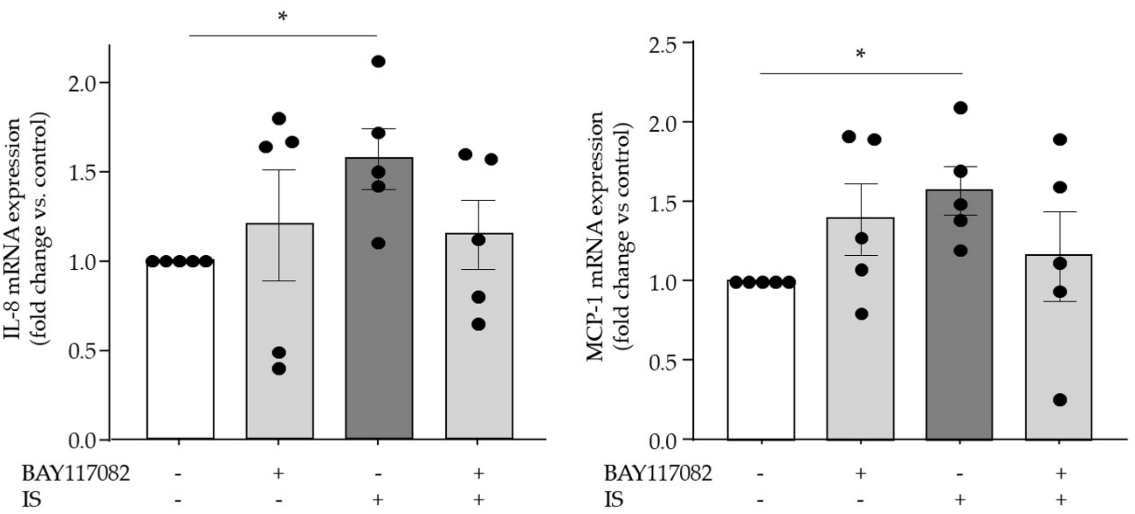

Supplemental Figure S3. Signaling pathways potentially involved in IL-8 and MCP-1 upregulation by indolic toxins

Non-activated AHR interacts with TAK1 and blocks TAK1-related p38 MAPK phosphorylation. AHR activation by indolic toxins induces AHR degradation by the proteasome leading to a decrease in AHR cytoplasmic expression. AHR decrease allows TAK1-related p38 MAPK phosphorylation. In turn, p38 induces JUN nuclear translocation that finally leads to the upregulation of IL-8 and MCP-1.

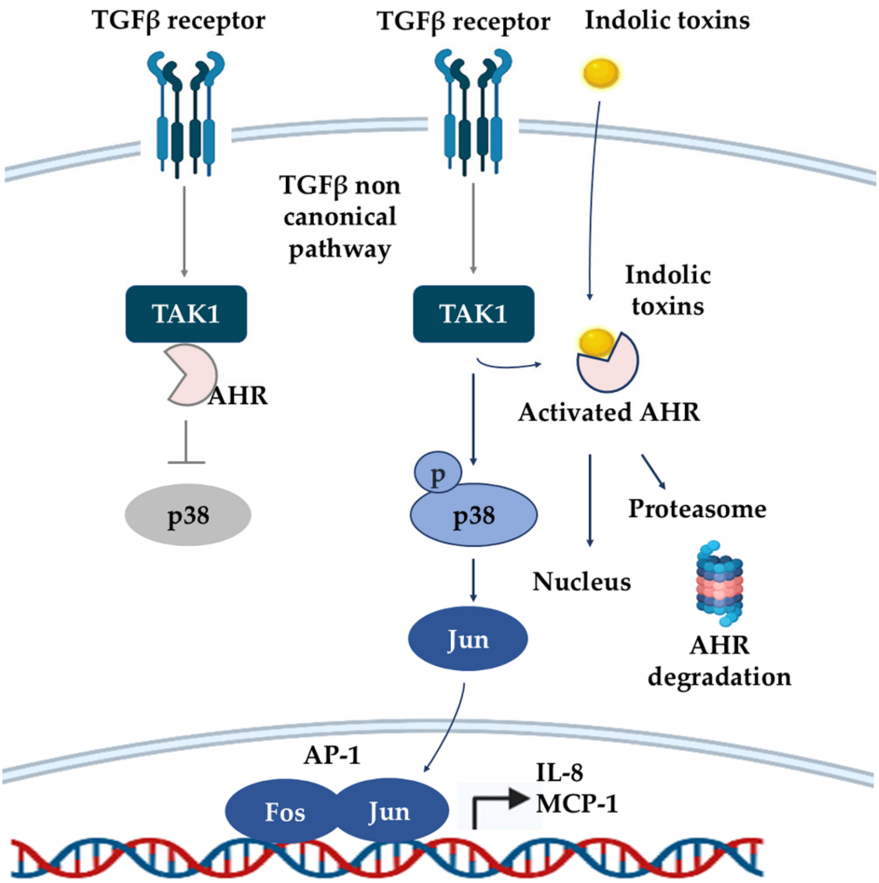

Supplement: Supplementary file 1 [file toxins-17-00434-s001.zip › toxins-3728558-supplementary.pdf]
